# Nerve ultrasound in amyotrophic lateral sclerosis: systematic review and meta-analysis

**DOI:** 10.1186/s42466-024-00346-z

**Published:** 2024-10-17

**Authors:** Ramy Abdelnaby, Ahmed Samy Shabib, Mostafa Hossam El Din Moawad, Talal Salem, Merna Wagih Youssef Awad, Peter Dawoud Awad, Imene Maallem, Hany Atwan, Salma Adel Rabie, Khaled Ashraf Mohamed, Hossam Abdelmageed, Ali M. Karkour, Mohamed Elsayed, Michael S. Cartwright

**Affiliations:** 1 Department of Neurology, RWTH Aachen University, Pauwels Street 30, 52074 Aachen, Germany; 2 Faculty of Medicine, Mansoura University, Mansoura, Egypt; 3 Faculty of Pharmacy Clinical Department, Alexandria University, Alexandria, Egypt; 4 Faculty of Medicine, Suez Canal University, Ismailia, Egypt; 5 Faculty of Medicine, Masaryk University, Brno, Czech Republic; 6 Faculty of medicine, Alexandria University, Alexandria, Egypt; 7 Department of Public Health, Theodor Bilharz Research Institute, Giza, Egypt; 8 Faculty of pharmacy, University Grenoble Alpes, La tronche, France; 9 Faculty of Medicine, Assiut University, Assiut, Egypt; 10 Faculty of Medicine, October 6 University, Giza, Egypt; 11 Faculty of medicine, Cairo University, Cairo, Egypt; 12 Neurology Department, University of Greifswald, Greifswald, Germany; 13 Microbiology Department, Faculty of Science, Tanta University, Tanta, Egypt; 14 Department of Psychiatry and Psychotherapy III, University of Ulm, Ulm, Germany; 15 Department of Psychiatry, School of Medicine and Health Sciences, Carl von Ossietzky University Oldenburg, Oldenburg, Germany; 16 Department of Neurology, Wake Forest School of Medicine, Winston-Salem, North Carolina USA

## Abstract

**Background/ Aim:**

Amyotrophic lateral sclerosis (ALS) is a neurodegenerative disease affecting upper and lower motor neurons, causing progressive atrophy of muscles, hypertonia, and paralysis. This study aimed to evaluate the current evidence and effectiveness of ultrasound in investigating nerve cross-sectional area (CSA) of peripheral nerves, vagus and cervical roots in those with ALS compared with healthy controls and to pool the CSA measurements.

**Methods:**

A systematic search was conducted on Cochrane, Clarivate Web of Science, PubMed, Scopus, and Embase for the mesh terms nerve, ultrasonography, and amyotrophic lateral sclerosis. A quality assessment was performed using the New-Ottawa scale. In addition, a double-arm meta-analysis using Review Manager 5 software version 5.4 was performed.

**Results:**

From the seventeen studies included in this review, the overall mean difference showed that individuals with ALS had a significantly smaller CSA in comparison to healthy controls for median, ulnar, C6 root, and phrenic nerves. However, no significant difference in the CSA was found in radial, vagal, sural, and tibial nerves.

**Discussion:**

This study confirmed results of some of the included studies regards the anatomic sites, where nerve atrophy in ALS could be detected to potentially support the diagnosis of ALS. However, we recommend further large, prospective studies to assess the diagnostic value of these anatomical sites for the diagnosis of ALS.

**Conclusions:**

Our findings confirmed specific anatomic sites to differentiate ALS patients from healthy controls through ultrasound. However, these findings cannot be used to confirm the ALS diagnosis, but rather assist in differentiating it from other diagnoses.

**Trial registration:**

Retrospectively registered on July 30th 2024 in PROSPERO (PROSPERO (york.ac.uk)) with ID574702.

**Graphical abstract:**

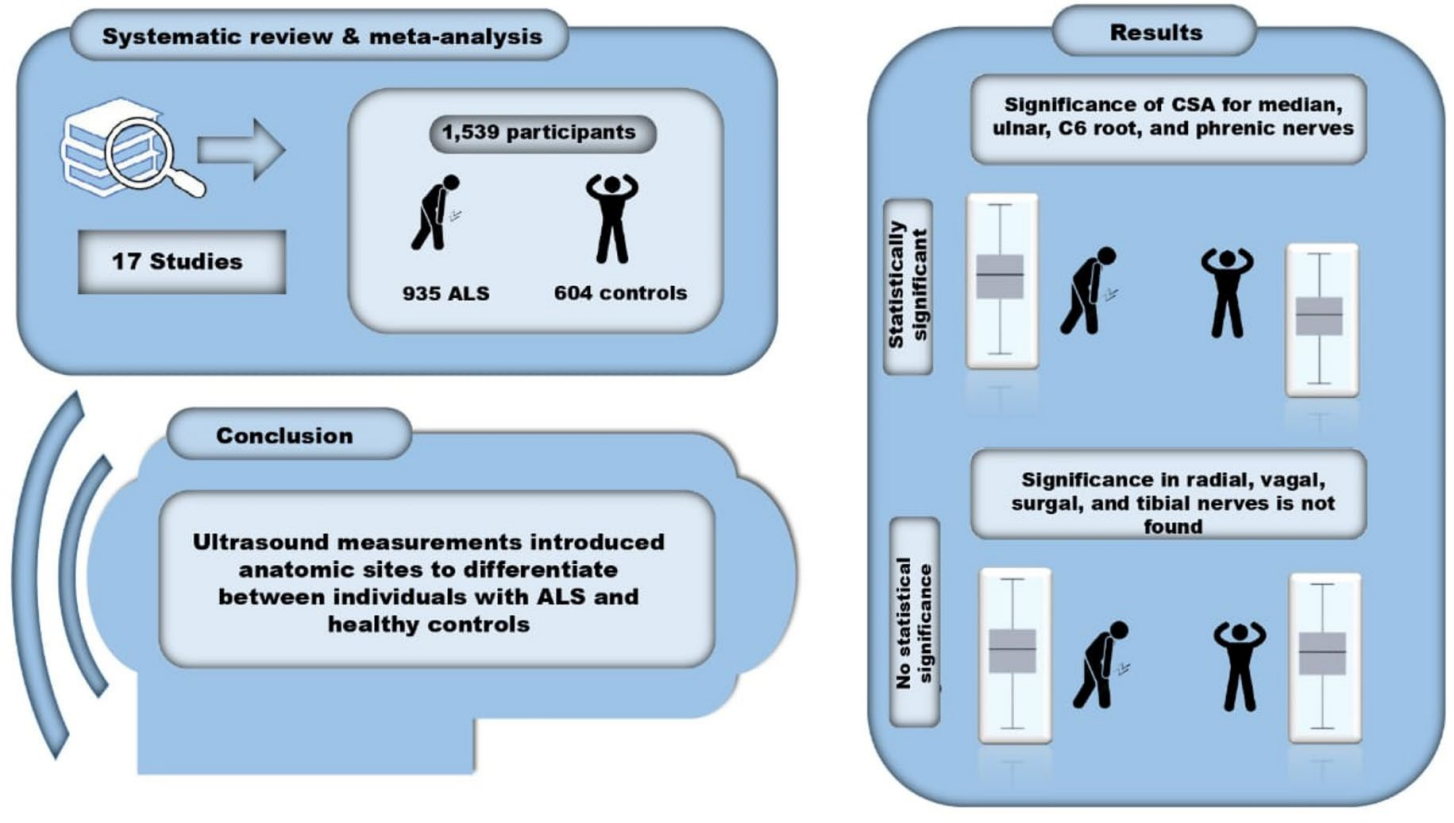

**Supplementary Information:**

The online version contains supplementary material available at 10.1186/s42466-024-00346-z.

## Background

Amyotrophic lateral sclerosis (ALS) is a neurodegenerative disease that affects both upper and lower motor neurons resulting in progressive muscular atrophy, spasticity, and paralysis. ALS remains a fatal disease with a prevalence and incidence of ALS are estimated to be 4.42 per 100 000 population and 1.59 per 100 000 person years, respectively [1]. Depending on the family history, ALS is classified as sporadic in 90% or familial in 10% of cases [2], and ALS appears to be mediated by complex molecular interactions. Despite novel treatments, patients with ALS have a median survival of only 3–4 years with an average age of onset between 58 and 60 years old [3].

The diagnosis of ALS was mainly clinical until the introduction of the revised El Escorial criteria in 2000 [4] and later the Awaji criteria [5] allowing the use of electromyography, though there were some concerns about the diagnostic sensitivity of these criteria. Recently, the Gold Coast criteria mentioned that the appropriate investigation depends on the clinical presentation and may include diagnostic tools such as electroneurophysiology and magnetic resonance imaging (MRI), or other imaging tools (i.e., it do not mention ultrasound (US) directly), but allows using it specifically to detect fasciculations in the diagnosis of ALS [6].

Nevertheless, the diagnosis of ALS remains challenging, especially at the early stages, due to the insidious nature of the disease [7]. Consequently, establishing more effective diagnostic methods is crucial to ensure the early diagnosis of ALS. It is now well established that muscle ultrasound is more sensitive for fasciculations than EMG. Multiple studies have reported nerve atrophy, as detected by reduced CSA, in ALS compared to mimicking disorders.

While that US should be interpreted in the context of electrophysiological diagnostic studies, which is indispensable in the ALS workup [8–12]. This could make the size of peripheral nerves as well as vagus or cervical roots, in addition to the clinical features, a potential diagnostic marker of ALS. Nerve US is a potentially diagnostic tool in ALS. Unfortunately, studies assessing nerve US suffer from heterogeneous findings and small sample sizes. Therefore, the aim of this meta-analysis is to assess the current evidence and significant difference in CSA of several peripheral nerves, vagus and cervical roots measured via US between ALS patients and healthy controls and to pool the CSA measurements.

## Methods

### Data sources & searches

**Fig. 1:**
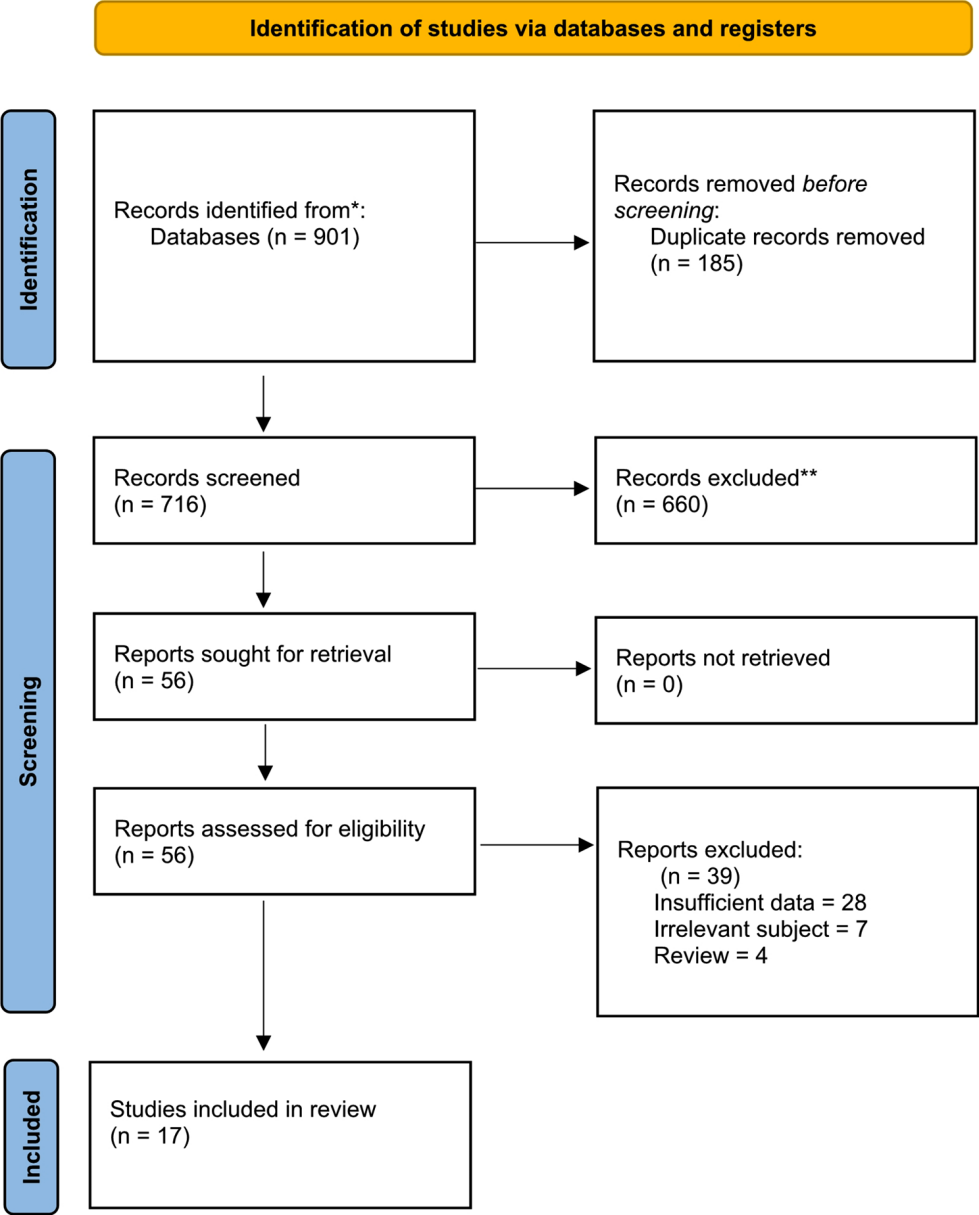
PRISMA flow diagram of the included studies

We conducted a systematic search of the following online databases by title and abstract from inception up to 29 July 2022: Cochrane, Clarivate Web of Science, PubMed, Scopus and Embase. The keywords of our search strategy were retrieved from the Medical Subject Headings terms (Mesh terms) for nerve, ultrasonography, and amyotrophic lateral sclerosis as follows: “Nerve” AND “Ultrasonography” OR “Ultrasound” OR “Ultrasonic” OR “Echotomography” OR “Sonography” OR “Sonographic” OR “Ultrasonographic” OR “Echography” AND “ALS” OR “Amyotrophic Lateral Sclerosis” OR “Gehrig Disease” OR “Motor Neuron Disease” OR “Lou Gehrig’s Disease”. Our study was conducted based on the Preferred Reporting Items for Systematic Reviews and Meta-Analyses (PRISMA) guidelines [13] and the methods described in the Cochrane Handbook [14]. Selection of the studies is illustrated in detail in the PRISMA flow diagram shown in Fig. 1.

### Eligibility criteria

We included studies published in international peer-reviewed journals, which included the following criteria: measuring the CSA of nerves in patients diagnosed with ALS by means of nerve ultrasonography. We included studies comparing ALS to healthy controls with no diseases. We excluded case reports, case series, letters to the editor, review articles, animal studies, studies that did not provide numerical measurements for CSA, and studies that used any imaging technique apart from nerve US. We excluded cases with other peripheral nerve disorders.

### Study selection and screening

Four authors [IM, HS, PD, SAR] screened records by title and abstract and then by full text according to the eligibility criteria to identify eligible studies. If there was no consensus regarding the eligibility of a study, a fifth author [RA] was consulted.

### Quality assessment

Two authors [MWY and PD] assessed the included papers separately in a blinded manner and any inconsistency was referred to a third author [KA]. We used the New-Ottawa scale (NOS) tool [15] for case-control studies, where studies scored 7–9 are considered of high, 4–6 of moderate, and 0–3 of low quality. The National Institutes of Health (NIH) tool [16] was used for cohort and cross-sectional studies, where the score of ≥ 7 is considered of good, 5–6 of fair, and < 5 of poor quality.

### Data analyses

We performed a double-arm meta-analysis using Review Manager 5 software version 5.4 to compare the mean CSA measurements of nerves in ALS patients with healthy controls by calculating the pooled mean difference of CSA. We conducted subgroup analyses by stratifying the studies according to the level of CSA measurement. Values that were reported as median, range, or interquartile range were converted to mean and standard deviation using the McGrath method. For studies presenting their measures using graphs, we extracted data using WebPlotDigitizer [17].

The random effect model of DerSimonian and Laird [18] was implemented to account for heterogeneity. Heterogeneity was assessed using Chi-squared tests and measured using I^2^ statistics. Heterogeneity was considered significant with I^2^ > 50%. P values < 0.05 were considered statistically significant. Sensitivity analyses were carried out in the form of leave-one-out analysis to examine the effect of elimination of each study on the overall results and it was conducted by Open Meta analyst software [19].

## Results

### Study selection and characteristics

Seventeen studies [9, 11, 12, 19–33] were included in the systematic review and meta-analysis., with a total of 935 ALS patients and 604 controls. The main characteristics of the included studies are reported in Table [S1].

### Quality assessment

From the seventeen studies included in this review, six [12, 22, 24, 25, 27, 32] were case-control, seven [9, 21, 23, 26, 29, 31, 33] were prospective cohort, two [20, 28] were retrospective cohort, and two [11, 30] were cross-sectional.

The results of the NOS and NIH tools are detailed in Table [S2] and Table [S3].

### Median nerve

**Fig. 2:**
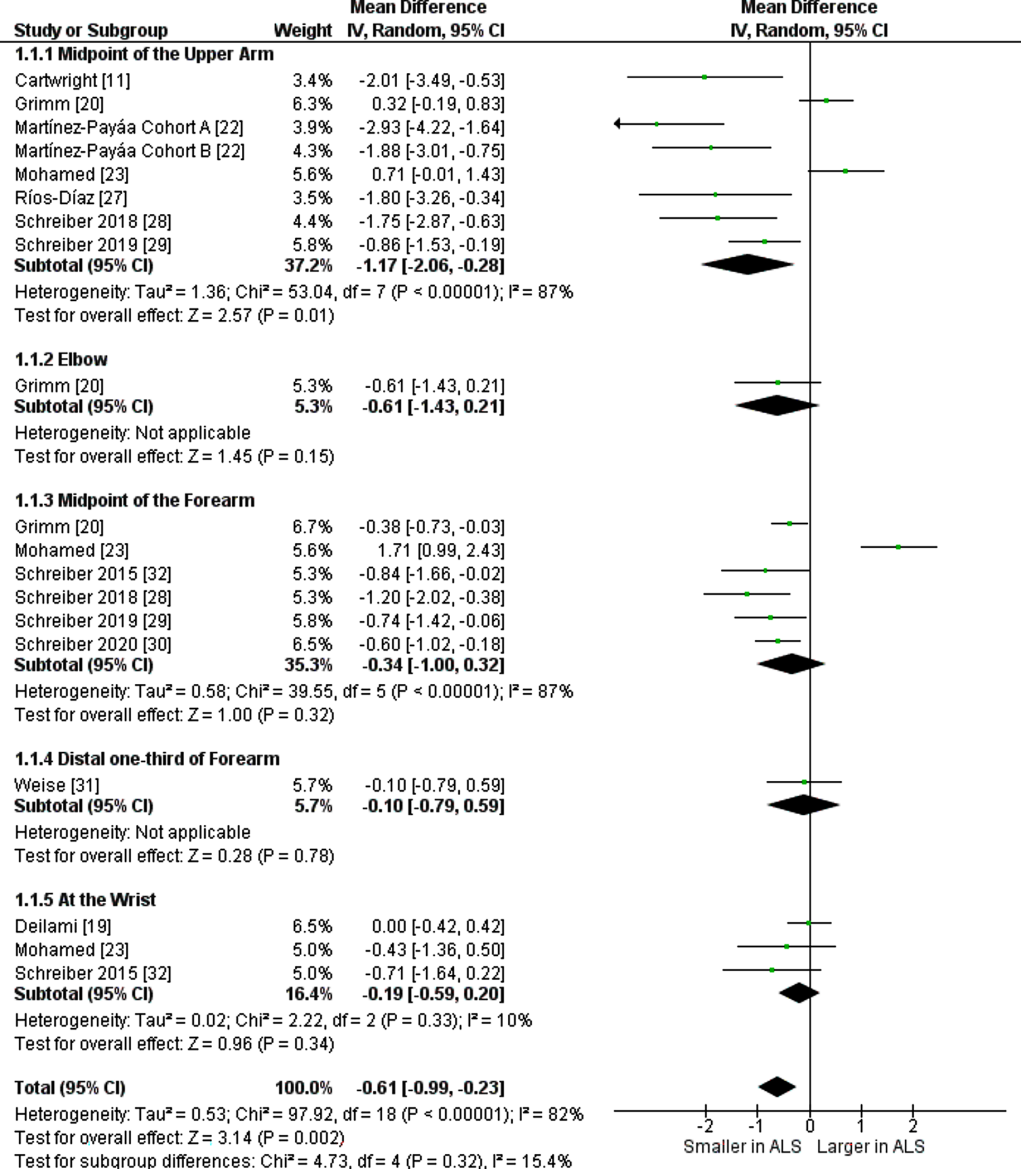
Mean difference [mm^2^] of bilateral median nerve cross sectional area between ALS patients and healthy controls. ALS: Amyotrophic Lateral Sclerosis, SD: Standard deviation, CI: Confidence Interval. *Cohort A comprised ALS patients diagnosed more than 6 months. *Cohort B comprised ALS patients with a recent (within 3 months) ALS diagnosis

Fourteen studies reported values of median nerve CSA at different levels from 1389 participants (860 ALS patients and 529 healthy controls), of which eleven studies reported the average measurement of bilateral median nerve and were sub-grouped according to the site of measurement. The analysis revealed a significant decrease in nerve CSA of ALS patients compared to healthy controls as detailed in Table 1; Fig. 2. The overall I^2^ test showed significant heterogeneity (I^2^ = 82%) and *p* > 0.00001.

Nine studies reported separate CSA values of right and left median nerve, and they were sub-grouped according to their levels of measurement. CSA of the median nerve was found to be significantly smaller in ALS patients than controls on both sides as shown in Tables 1 and 2, and 3 and in Figure S1 and S2. Heterogeneity was significant by I^2^ = 73% (*p* < 0.00001) and I^2^ = 71% (*p* = 0.0005) for right and left sides respectively.

**Table 1:** Sonographic cross-sectional area (mean ± SD) of average bilateral median nerve

Study ID	ALS			Controls		
Midpoint of the Upper Arm	Mean [mm^2^]	SD [mm^2^]	N	Mean [mm^2^]	SD [mm^2^]	N
Cartwright [11]	10.7	2.6	20	12.7	2.1	20
Grimm [20]	9.3	0.98	17	8.99	0.56	28
Martínez-Payáa Cohort A [22]	8.32	2.49	27	11.25	3.04	46
Martínez-Payáa Cohort B [22]	9.37	2.7	57	11.25	3.04	46
Mohamed [23]	6.8	1.95	30	6.1	1.0	100
Ríos-Díaz [27]	9.2	2.7	59	11.0	2.9	20
Schreiber 2018 [28]	9.25	1.5	41	11.0	2.2	18
Schreiber 2019 [29]	9.24	1.7	113	10.1	1.7	32
Elbow						
Grimm [20]	9.09	1.6	17	9.7	0.85	28
Midpoint of the forearm						
Grimm [20]	6.8	0.6	17	7.19	0.48	28
Mohamed [23]	6.5	1.9	30	4.8	0.9	100
Schreiber 2015 [32]	7.06	1.7	70	7.9	1.5	18
Schreiber 2018 [28]	7.4	1.45	41	8.6	1.5	18
Schreiber 2019 [29]	7.4	1.7	171	8.17	1.86	34
Schreiber 2020 [30]	8.0	1.4	177	8.6	1.4	57
Distal one-third of Forearm						
Weise [31]	5.7	1.5	37	5.8	1.5	40
At the Wrist						
Deilami [19]	5.0	0.9	35	5.0	0.9	35
Mohamed [23]	6.7	2.5	30	7.2	1.0	100
Schreiber 2015 [32]	9.49	1.78	70	10.2	1.8	18
**Total**			1059			786

**Table 2:** Sonographic cross-sectional area (mean ± SD) of right median nerve

Study ID	ALS			Controls		
Midpoint of the Upper Arm	Mean [mm^2^]	SD [mm^2^]	N	Mean [mm^2^]	SD [mm^2^]	N
Martínez-Payáa Cohort A [22]	8.3	2.3	27	11.3	2.98	46
Martínez-Payáa Cohort B [22]	9.5	2.83	57	11.3	2.98	46
Noto [25]	7.9	1.3	53	9.0	1.4	30
Schreiber 2018 [28]	9.3	1.5	41	10.8	2.1	18
Midpoint of the forearm						
Mori [24]	7.4	2.3	21	6.8	1.5	30
Noto [25]	6.2	1.2	53	6.2	0.8	30
Schreiber 2015 [32]	7.15	1.8	70	8.1	1.6	18
Schreiber 2018 [28]	7.4	1.2	41	8.6	1.5	18
Distal one-third of Forearm						
Weise [31]	5.6	1.4	37	5.7	1.6	40
At the Wrist						
Deilami [19]	5.3	1.0	35	5.5	1.0	35
Mori [24]	8.1	2.0	21	8.8	2.1	30
Nodera [8]	5.7	1.5	35	6.7	1.2	37
Noto [25]	10.2	1.7	53	10.1	1.6	30
Schreiber 2015 [32]	9.47	1.78	70	10.0	1.9	18
**Total**			614			426

**Table 3:** Sonographic cross-sectional area (mean ± SD) of left median nerve

Study ID	ALS			Controls		
Midpoint of the Upper Arm	Mean [mm^2^]	SD [mm^2^]	N	Mean [mm^2^]	SD [mm^2^]	N
Cartwright [11]	10.4	2.5	20	12.5	1.68	20
Martínez-Payáa Cohort A [22]	8.3	2.6	27	11.17	3.1	46
Martínez-Payáa Cohort B [22]	9.2	2.6	57	11.17	3.1	46
Schreiber 2018 [28]	9.2	1.5	41	11.2	2.3	18
Midpoint of the forearm						
Schreiber 2015 [32]	6.9	1.67	70	7.7	1.5	18
Schreiber 2018 [28]	7.4	1.7	41	8.6	1.5	18
Distal one-third of Forearm						
Weise [31]	5.8	1.7	37	5.9	1.5	40
At the Wrist						
Deilami [19]	5.2	1.0	35	5.6	1.0	35
Schreiber 2015 [32]	9.5	1.77	70	10.4	1.7	18
**Total**			398			259

We conducted a subgroup analysis by disease duration, ultrasound probe frequency, age, and ALS functional rating scale (ALSFR) score for assessment of sources of heterogeneity and these variables effect on collected measurements. Subgroup analysis by disease duration, ultrasound probe frequency, and age included 741 ALS patients and 462 healthy controls and revealed a significant difference in CSA with mean difference (MD) = -0.8 (95% CI: -1.26, -0.35, *p* < 0.00001). Subgroup analysis by ALSFR included 724 ALS patients and 434 healthy controls with CSA MD = -0.89 (95% CI: -1.39, -0.38, *p* < 0.00001). This is detailed in supplementary Figs. 3, 4, 5, and 6.

### Ulnar nerve

**Fig. 3:**
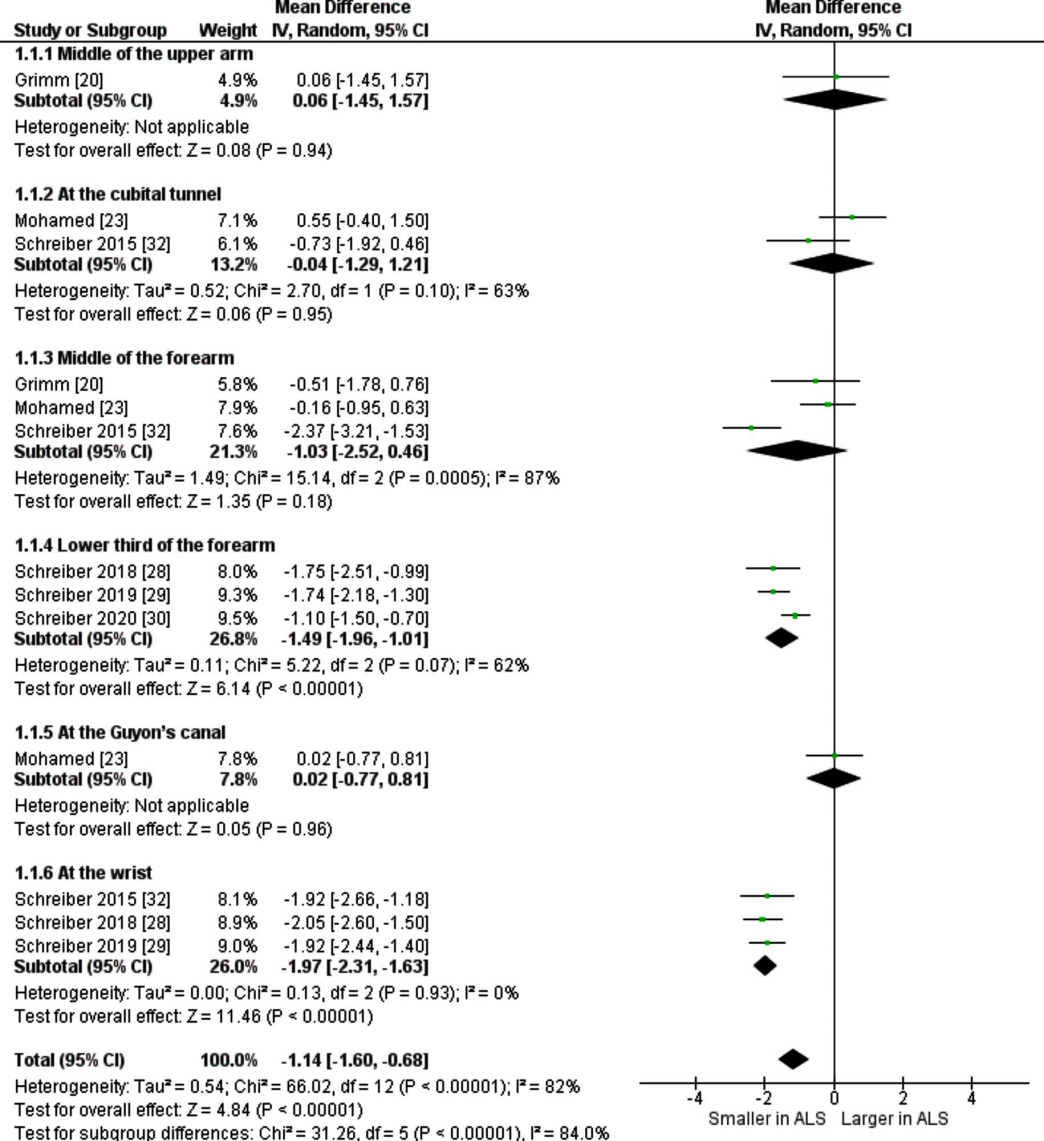
Mean difference [mm^2^] of bilateral ulnar nerve cross sectional area between ALS patients and healthy controls

Nine studies reported values of ulnar nerve CSA at separate anatomical sites: mid-upper arm, at the cubital tunnel, mid-forearm, lower third of the forearm, at Guyon’s canal, and at the wrist (just proximal to Guyon’s canal), including six studies described average measurements of bilateral ulnar nerve in both ALS patients and controls which were stratified into six groups according to their level of measurement. CSA of ALS patients was found to be significantly smaller than CSA of healthy controls at lower third of forearm, wrist and total and the main findings are detailed in Table 4; Fig. 3.

**Table 4:** Sonographic cross-sectional area (mean ± SD) of average bilateral ulnar nerve

Study ID	ALS			Controls		
Midpoint of the Upper Arm	Mean [mm^2^]	SD [mm^2^]	N	Mean [mm^2^]	SD [mm^2^]	N
Grimm [20]	7.17	2.09	17	7.1	3.07	28
At the cubital tunnel						
Mohamed [23]	7.0	2.46	30	6.45	1.0	30
Schreiber 2015 [32]	7.1	1.89	70	7.8	2.4	18
Midpoint of the forearm						
Grimm [20]	5.6	2.32	17	6.1	1.69	28
Mohamed [23]	6.14	1.9	30	6.3	1.1	30
Schreiber 2015 [32]	4.78	1.49	70	7.15	1.65	18
Lower third of Forearm						
Schreiber 2018 [28]	5.5	1.2	41	7.3	1.4	18
Schreiber 2019 [29]	5.26	1.29	165	7.0	1.3	41
Schreiber 2020 [30]	5.9	1.4	177	7.0	1.3	57
At the Guyon’s canal						
Mohamed [23]	5.92	1.8	30	5.9	1.3	30
At the wrist						
Schreiber 2015 [32]	4.18	1.35	70	6.1	1.45	18
Schreiber 2018 [28]	4.45	0.95	41	6.5	1.0	18
Schreiber 2019 [29]	4.49	1.18	164	6.41	1.57	39
**Total**			922			373

Five studies provided numerical measures for right ulnar nerve CSA, and they were sub-grouped according to the previously mentioned sites. CSA in ALS patients was − 1.15 mm^2^ less than in controls as detailed in Table 5 and Figure S7. Heterogeneity (I^2^ = 89%, *p* > 0.00001) was statistically significant.

**Table 5:** Sonographic cross-sectional area (mean ± SD) of right ulnar nerve

Study ID	ALS			Controls		
Middle of the upper arm	Mean [mm^2^]	SD [mm^2^]	N	Mean [mm^2^]	SD [mm^2^]	N
Noto [25]	6.7	1.3	53	6.8	1.2	30
At the cubital tunnel						
Schreiber 2015 [32]	7.03	2.24	70	8.1	2.4	18
Middle of the forearm						
Mori [24]	6.4	1.8	21	7.2	2.2	30
Noto [25]	5.7	1.0	53	5.9	1.4	30
Schreiber 2015 [32]	4.6	1.4	70	7.4	1.6	18
Lower third of Forearm						
Schreiber 2018 [28]	5.6	1.2	41	7.6	1.5	18
At the Guyon’s canal						
Mori [24]	5.2	1.5	21	5.1	1.0	30
Nodera [8]	4.1	1.3	35	5.3	1.3	37
At the wrist						
Noto [25]	5.7	1.2	53	5.9	1.3	30
Schreiber 2015 [32]	4.07	1.4	70	6.1	1.5	18
Schreiber 2018 [28]	4.4	0.9	41	6.8	1.0	18
**Total**			528			277

Schreiber et al. 2018 [29] and Schreiber et al. 2015 [33] reported measurements of left ulnar nerve in both ALS patients and healthy controls. Subgroup analysis by level of measurement was implemented and revealed a significant decrease in nerve CSA of ALS patients compared to controls at middle of the forearm, lower third of forearm, wrist and total. This is detailed in Table 6 and Figure S8. Heterogeneity was insignificant with I^2^ = 24% and *p* = 0.26.

**Table 6:** Sonographic cross-sectional area (mean ± SD) of left ulnar nerve

Study ID	ALS			Controls		
At the cubital tunnel						
Schreiber 2015 [32]	7.2	1.54	70	7.6	2.4	18
Middle of the forearm						
Schreiber 2015 [32]	4.95	1.54	70	6.9	1.7	18
Lower third of Forearm						
Schreiber 2018 [28]	5.5	1.2	41	7.0	1.4	18
At the wrist						
Schreiber 2018 [28]	4.5	1.0	41	6.2	1.0	18
Schreiber 2015 [32]	4.29	1.26	70	6.1	1.4	18
**Total**			292			90

A subgroup analysis by disease duration, age, and ALSFR score was performed for ulnar nerve measurements. Subgroup analysis by disease duration and age included 500 ALS patients and 192 healthy controls and revealed a significant difference in CSA with MD = -1.18 (95% CI: -1.75, -0.61, *p* = 0.0005). Subgroup analysis by ALSFR included 483 ALS patients and 164 healthy controls with CSA MD = -1.29 (95% CI: -1.88, -0.69, *p* = 0.0006). This is shown in supplementary Figs. 9, 10 and 11.

### Other nerves

#### Vagus nerve

Four studies provided measurements of the average bilateral vagus nerve for ALS patients and control groups, and they were sub-grouped at two sites; the carotid bifurcation and the thyroid gland; moreover, three of these studies provided measurements of vagal nerve on right and left sides at the level of thyroid gland. Analysis of average bilateral, right and left vagal nerve showed insignificant difference in nerve CSA between ALS patients and controls and this is detailed in Table 7 and Figures S12 (A, B, C). Heterogeneity was significant with I^2^ = 89% and *p* > 0.00001; I^2^ = 92% and *p* > 0.00001; I2 = 89% and *p* = 0.0001 for average bilateral, right, and left sides respectively.

**Table 7:** Sonographic cross-sectional area (mean ± SD) of average bilateral vagal nervs

Study ID	ALS			Controls		
	Mean [mm^2^]	SD [mm^2^]	*N*	Mean [mm^2^]	SD [mm^2^]	*N*
Carotid bifurcation						
Grimm [20]	1.7	0.92	17	2.49	0.28	28
Thyroid gland						
Holzapfel [21]	1.85	0.65	24	2.1	0.45	19
Papadopoulou [26]	1.6	0.58	21	2.6	0.8	28
Weise [31]	1.55	0.5	37	1.5	0.5	40
**Total**			99			115

Data were available to conduct a subgroup analysis by disease duration, US probe frequency, and age for vagal nerve measurements which included 96 ALS patients and 124 healthy controls and revealed a significant difference in CSA with MD = -0.67 (95% CI: -1.29, -0.06, *p* < 0.00001) as shown in Supplementary Fig. 13 (A, B, C).

Radial nerve: Two studies reported CSA values of the radial nerve at the spiral grove, with 289 measurements (71 ALS patients and 218 controls). The overall mean difference was found to be insignificant between both groups and the main findings are detailed in Table 8 and Figure S14 (A). There was significant heterogeneity with I^2^ = 78% and *p* = 0.03.

**Table 8:** Sonographic cross-sectional area (mean ± SD) of radial nerve at spiral groove

Study ID	ALS			Controls		
	Mean [mm^2^]	SD [mm^2^]	*N*	Mean [mm^2^]	SD [mm^2^]	*N*
Mohamed [23]	5.05	2.06	30	5.08	1.2	200
Schreiber 2018 [28]	5.7	1.2	41	6.8	1.3	18
**Total**			71			218

#### Tibial nerve

Two studies provided numerical values for tibial nerve CSA with 288 total measurements (94 ALS patients and 194 controls). A subgroup analysis was carried out based on the level of nerve measurement: at the popliteal fossa and at the ankle joint. There was no significant difference in nerve CSA between both groups and the main findings are presented in Table 9 and Figure S14 (B). Heterogeneity was significant with I^2^ = 83% and *p* = 0.0006.

**Table 9:** Sonographic cross-sectional area (mean ± SD) of tibial nerve

Study ID	ALS			Controls		
	Mean [mm^2^]	SD [mm^2^]	*N*	Mean [mm^2^]	SD [mm^2^]	*N*
At the popliteal fossa						
Grimm [20]	23.6	5.1	17	21.45	4.1	28
Mohamed [23]	14.09	5.1	30	19.0	6.9	69
At the ankle joint						
Grimm [20]	9.02	2.55	17	9.16	2.04	28
Mohamed [23]	9.9	3.34	30	12.0	4.5	69
**Total**			94			194

#### Sural nerve

Two studies described values for the average bilateral sural nerve CSA, with 85 total measurements (37 ALS patients and 48 healthy controls). There was no significant between ALS patients and healthy controls, and no significant heterogeneity was detected with I^2^ = 14% and *p* = 0.28, as detailed in Table 10 and Figure S14 (C).

**Table 10:** Sonographic cross-sectional area (mean ± SD) of sural nerve

Study ID	ALS			Controls		
	Mean [mm^2^]	SD [mm^2^]	*N*	Mean [mm^2^]	SD [mm^2^]	*N*
Cartwright [11]	4.5	1.2	20	5.2	1.5	20
Grimm [20]	1.98	0.6	17	2.18	0.5	28
**Total**			37			48

Other nerve CSA measures were reported by single studies and implementing a meta-analysis was not possible. Nodera et al. [9] reported separate measures of C6 root CSA for both ALS patients and controls. ALS patients were found to have smaller CSA compared to healthy controls with a significant mean difference equals 2.26 mm^2^ between both measures.

Suratos et al. [11] compared values of phrenic nerve CSA between ALS patients and healthy controls. A significant decrease in CSA was found in ALS nerve measures on both sides. Nerve measurements are detailed in Table S4.

### Heterogeneity

Heterogeneity was found among studies measuring vagus, median, ulnar, radial, and tibial nerves. Causes of heterogeneity can be attributed to clinical variation between study samples, difference in disease severity between patients, variable methods for diagnosis and assessment. Therefore, subgroup analyses and sensitivity analyses were conducted to detect if any study represents a major source of heterogeneity.

### Sensitivity analysis

#### Median nerve

One study [24] compared results to the measurements of another study reporting reference values of the median nerve. Removing two studies [21, 24] from the combined results of average median nerve led to a decrease in I^2^ from 87 to 47% and from 87 to 1% at the upper arm and forearm subgroups respectively and overall MD increased from − 61 mm^2^ to -0.85 mm^2^. This shows that heterogeneity became statistically insignificant as shown in Figure S15.

Another study [23] showed major clinical heterogeneity, which may affect the end results of right-sided analysis. On removal of this study from the upper arm subgroup, I^2^ decreased from 62% to zero with an overall mean − 0.6 mm^2^ (95% CI: -0.94, -0.25, *p* = 0.00003) and overall MD decreased from − 0.74 mm^2^ to -0.6 mm^2^ (Figure S16).

#### Ulnar nerve

Schreiber et al. 2015 [33], who included variable ALS phenotypes, presented a major source of heterogeneity in the middle forearm subgroup of both right and bilateral ulnar nerve analyses. Elimination of this study caused I^2^ to decrease from 87% to zero and from 92% to zero in bilateral nerve analysis and right-side, respectively, as shown in Figures S17 (A), (B).

One study [26] was removed from the wrist subgroup of right-sided analysis to resolve the significant heterogeneity. Its elimination caused I^2^ to decrease from 94% to zero and MD increased from − 1.54 mm^2^ to -2.28 mm^2^, indicating it was a major source of heterogeneity Figure S17 (C).

## Discussion

The overall mean differences revealed that ALS patients showed significantly smaller CSAs in comparison to healthy controls for the median and ulnar nerves. Conversely, no significant differences in CSAs were detected for the radial, vagus, sural, and tibial nerves between ALS patients and healthy controls. However, the small difference in the mean difference was statistically significant but needs to be interpreted and used clinically with caution as for the few millimeters difference in the effect size (mean difference) wouldn’t make a difference clinically hence clinically insignificant in the diagnosis of ALS. However, combining the nerve ultrasound data with muscle US parameters increased the diagnostic sensitivity and therefore the utility of the nerve US in ALS Diagnosis [28].

The non-significant findings in the radial and tibial nerves can be attributed to their CSA being correlated with factors such as height, weight, gender, and BMI [21, 24, 29]. Thus, controlling these variables is essential to draw a well-founded conclusion regarding these nerves. Furthermore, the non-significant difference in CSA for the sural nerve was expected, as it is primarily a sensory nerve typically unaffected by ALS. Additionally, the vagus nerve, being relatively small, presents a challenge for clinicians in detecting subtle CSA changes.

Due to the compensatory reinnervation seen in ALS patients, muscle weakness does not clinically manifest until a significant number of motor neurons are lost [34]. Additionally, motor nerve conduction studies can appear normal in the early stages of ALS [35]. Moreover, as nerve atrophy precedes ALS clinical manifestations [36], there is a need for a sensitive ALS diagnostic tool in the early stages of the disease. While the utility of US in detecting muscle fasciculations is more useful, ultrasonography of peripheral nerves is an evolving and non-invasive tool that can help to detect early axonal loss in suspected ALS patients as well as in the diagnosis and differentiation of ALS from its mimic diseases [21, 37]. Therefore, it is important to acknowledge the need for prospective studies on suspected ALS patients in order to establish this comparison.

We found that the median nerve CSA at the mid-arm showed the largest reduction in CSA when comparing ALS patients to healthy controls. This suggests this might be the most sensitive anatomical site for observing the atrophic changes in peripheral nerves in ALS patients.

We found significant heterogeneity among the studies measuring the vagus, median, ulnar, radial, and tibial nerves. This suggests that there is considerable variability between the included studies, particularly regarding the clinical characteristics of the patients and ALS-phenotypes, duration of the disease, site of onset, ALSFRS score. Furthermore, other sources contributing to increased heterogeneity include different scanning protocols, utilization of different US probes, and variations in the sites selected for nerve measurements.

To investigate the effect of these variables, we conducted a subgroup analysis according to disease duration, US probe frequency, age and ALSFR score for the median and ulnar nerve. The test for subgroup differences in median nerve subgroup analyses revealed that there is no significant subgroup effect, *p* = 0.7, 0.27, 0.87, and 0.32 in disease duration, US probe frequency, age, and ALSFR respectively. The test for subgroup differences in subgroup analysis by age for ulnar nerve indicated that there is a statistically significant difference between both groups (*p* = 0.0002) with smaller CSA in patients with disease duration more than 25 months suggesting progressive ulnar nerve atrophy with greater disease duration. Finally, the test for subgroup differences in ulnar nerve subgroup analyses by age and ALSFR revealed that there is no significant subgroup effect (Supplementary Figs. 3–6 and 9–11).

One study [23] was a major source of clinical heterogeneity, with severe and variable disease severity (mean ALSFR score = 25.67 ± 11.05), compared to the other included studies.

This meta-analysis has limitations. First, the low number of studies found for the radial, vagus, tibial, sural as well as the lack of disease controls in those studies. Second, included studies did not mention if the site of ALS onset or the dominant hand had more nerve atrophy and did not control for confounders that could affect nerve measurements such as age, height and BMI and this needs further research. Additionally, the high heterogeneity of the data collected from the included studies, which resolved after using sensitivity analysis, still remains a concern. Third, it’s important to note that our meta-analysis does not provide specific recommendations regarding the optimal timing for detecting nerve atrophic changes. Also, the included studies compare ALS with healthy controls not disease mimics limiting the strength of diagnostic evidence.

The diagnostic accuracy of ALS utilizing nerve CSA alone can be as low as 72.6%. This can be increased by combing nerve CSA with muscle US parameters [27]. However, we were limited by the low number of studies in the literature looking at muscle biomarkers in ALS patients. Hence, more studies are needed in this regard.

While the difference in CSA between healthy control subjects and ALS patients did reach statistical significance in certain nerves, it’s important to note that this difference was relatively minimal, measuring around 1 mm². This raises concerns regarding the clinical applicability of these findings, particularly considering the limitations of US difference in US device specifications and protocols and the potential for inter-observer variability in measurements.

The Gold Coast criteria do not mention using nerve CSA to help in ALS diagnosis. This is expected given the fact that performing muscle ultrasound in ALS is more useful and could be so far more important detecting the fasciculations than nerve CSA based on the current evidence. As a result, one cannot depend on nerve CSA as measured by US as evidence of LMNL in the criteria and this limit the applicability of this meta-analysis [6].

It’s also worth highlighting the absence of a universally accepted consensus regarding a definitive cutoff point below which a CSA can be unequivocally classified as abnormal. Since it is difficult to differentiate between ALS and its mimicking disorders with LMN dysfunction through nerve CSA measurements, nerve CSA can be useful in differentiating ALS from other diagnoses.

We recommend establishing a standardized ultrasonographic imaging protocol to obtain nerve CSAs, along with considering the variability of clinical phenotypes of ALS patients as well as the stage of the disease (as indicated by the ALSFRS-R score).

## Conclusions

Our findings confirmed specific anatomic sites (the median nerve at the mid-arm and the ulnar nerve at the wrist and the lower third of the forearm) to differentiate ALS patients with smaller CSA in comparison to healthy controls when using nerve US. However, these findings cannot be used to confirm the ALS diagnosis, but rather assist in differentiating it from other diagnoses. Nevertheless, further research with larger, better quality prospective diagnostic cohorts is required to assess their diagnostic value in ALS patients. This distinction may serve as a biomarker at a group level for further monitoring.

## Electronic supplementary material

Below is the link to the electronic supplementary material.

Supplementary Material 1

## Data Availability

The datasets used and/or analyzed during the current study are available from the corresponding author on reasonable request.

## Abbreviations

ALS: Amyotrophic Lateral Sclerosis
CSA: Cross Sectional Area
MRI: Magnetic Resonance Imaging
US: Ultrasound
Mesh terms: Medical Subject Headings terms
PRISMA: Preferred Reporting Items for Systematic Reviews and Meta-Analyses
NOS: New-Ottawa Scale
NIH: National Institutes of Health
ALSFR: ALS Functional Rating Scale
