# Supplementary material for: Nerve ultrasound in amyotrophic lateral sclerosis: systematic review and meta-analysis
Source: Neurol Res Pract. 2024 Oct 17;6:47. doi: 10.1186/s42466-024-00346-z (PMC11484457; doi:10.1186/s42466-024-00346-z)
Supplement: Supplementary file 1 — Supplementary Material 1 [file 42466_2024_346_MOESM1_ESM.docx]

**Table S1. Characteristics of studies included in meta-analysis**

| **Study (Author, Ref)** | **Country** | **Study design** | **Participants (disease, N)** | | **Sex** | | **Age** | | **ALSFRS-R** | **Nerves and Site of measurement** | **Ultrasound device** | **Method of measurement** | **ALS diagnosis** | **ALS duration (months) and phenotype (N, (%))** |
| --- | --- | --- | --- | --- | --- | --- | --- | --- | --- | --- | --- | --- | --- | --- |
|  |  |  | Cases | Control | Cases (M/F) | Control (M/F) | Cases | Control |  |  |  |  |  |  |
| Cartwright [11] | USA | Case-control | 20 | 20 | 10/10 | 10/10 | 58 (40-71) | 58.1 (42-76) | 30.5 ± 9 | Median:  Midpoint of the arm.  Sural:  10 cm above the lateral malleolus | A Biosound MyLab 25 (Esaote Group, Genoa, Italy) with an 18 MHz linear array transducer. | Bilateral measurement in a supine or seated position, with the technician facing the patient. | Patients were recruited based on the revised El Escorial criteria.  ALS diagnosed by experienced ALS clinicians and each participant had extremity strength testing.  ALS assessed using the forced vital capacity (FVC) and the ALS Functional Rating Scale (ALSFRS). | Duration: 25.1 ± 14.47  Clinical onset:  NA |
| Deilami [19] | Iran | Cross-sectional | 35 | 35 | 19/16 | 22/13 | 39.2±14 | 38±11 | 36.5±5.3. | Median:  Between the flexor digitorum superficialis and flexor digitorum profundus muscles. | SonoSite M-Turbo with an 8-18 Hz linear probe. | In a seated position with straight and supinated arms. | The revised El Escorial criteria and Awaji diagnostic criteria to recruit the ALS patients.  All patients and healthy controls underwent neurological examination, and values of ALSFRS were recorded. | Duration: 4.43 ± 1.1 (years).  Clinical onset:  NA |
| Grimm [20] | Germany | Prospective cohort | 17 | 28 | 9/6 | 17/13 | 65.6 ± 13.3 | 52.8 ± 17.2 | - | Median:  In the upper arm next to the brachial artery, in the middle of the forearm.  Ulnar:  In the upper arm and at the mid-forearm.  Tibial:  In popliteal space (proximal) and at medial malleolus.  Fibular:  2 cm above the fibular head.  Sural:  Between the lateral and medial head of the gastrocnemic muscle. | A high frequency 14 MHz probe real-time linear array scanner (ZONARE Ultrasound systems). | In axial planes and the cross-sectional area (CSA) was measured at standardized anatomical points.  Upper and lower limbs and in the neck was measured bilaterally. | The revised El Escorial criteria and Awaji diagnostic criteria to diagnose ALS according to clinical signs and electromyography. | Duration: 18.8 ± 18.3  Clinical onset:  Arm: 7  Leg: 6  Bulbar: 4 |
| Holzapfel [21] | Germany | Case-control | 24 | 19 | 16/8 | 13/6 | 64.04 ± 9.98 | 63.05 ± 11.07 | 41.63 ± 5.03 | the right and left vagus nerves were measured at the level of the thyroid gland in the carotid sheath | Philips IU22 with a 17-MHz linear array transducer. | The nerves were scanned in the supine position in axial planes. | The Awaji criteria to classify the patients by clinical, electrophysiological, and ultrasound examinations.  The ALS Functional Rating Scale (ALS-FRS) to evaluate the functional status of patients with ALS.  The bulbar subscale of the ALSFRS-R assessing bulbar impairment. | Duration: 12.46 ± 10.28  Clinical onset:  Bulbar: 10 (41.7) |
| Paya [22] | Spain | Prospective cohort | Cohort A: 27 Cohort B: 57 | 46 | Cohort A: 18/9 Cohort B: 33/24 | 27/19 | Cohort A: 58.7 ± 8.2 Cohort B: 65.1 ± 8.8 | 59.9 ± 8.08 | Cohort A: 25.67 ± 11.05 Cohort B: 38.7 ± 5.25 | Median:  Bilaterally at midpoint of the arm between the medial epicondyle and the axilla. | General Electric Company LEbt12 (cohort A) with a linear transducer at 5—13 MHz.  Canon Medical Systems Toshiba Aplio XG (cohort B) with a linear transducer at 7—13MHz. | Participants were assessed in supine position with the arm supinated and abducted beside the body. | The Awaji criteria to recruit the patients with a clinical diagnosis of probable or definitive ALS.  The Revised Amyotrophic Lateral Sclerosis Functional Rating Scale (ALSFRS-R) to assess the disability. | Cohort A (n = 27)  Duration:33.30 ± 28.55  Clinical onset:  Bulbar: 8 (29.63)  Lower limbs: 12 (44.44)  Upper limbs: 7 (25.93)  Cohort B (n = 57)  Duration: 15.65 ± 11.62  Clinical onset:  Bulbar: 14 (24.56)  Lower limbs: 27 (47.37)  Upper limbs: 16 (28.07) |
| Mohamed [23] | Egypt | Case-control | 30 | 100 | 21/9 | 50/50 | 43.5 ± 16 | 39 ± 14 | 37.2±8.4 | Median:  At the carpal tunnel, mid-forearm and the upper arm.  Ulnar:  At the Guyon’s canal, at the mid-forearm and the upper arm. Radial:  At the spiral grove.  Posterior tibial:  At the tarsal tunnel.  Tibial:  At the popliteal fossa. | Ogiq p7 (GE healthcare, Waukesha, Wisconsin, USA) with a linear transduced 7–12 MHz. | - | The El Escorial World Federation of Neurology Criteria for the Diagnosis of ALS.  The Revised Amyotrophic Lateral Sclerosis Functional Rating Scale (ALSFRS-R) to assess the disability | Duration: 1.8 ± 1.4  (years)  Clinical phenotype:  Bulbar: 2 (6.7)  Classic: 19 (63.3)  Classic/familial: 7 (23.3)  Flail chest: 1 (3.3)  Local segment: 1 (3.3) |
| Mori [24] | Japan | Case-control | 21 | 30 | 17/4 | 20/10 | 68.3 ± 9.1 | 63.9 ± 16.8 | - | The right median and ulnar: At the wrist, 2 cm proximal to the wrist crease and in the mid forearm. | LOGIQ7 (GE) with an 11-MHz linear array transducer. | Single and blinded technician measure in supine position.  The (CSA) were measured inside the hyperechoic rim (epineurium). | The revised El Escorial criteria and Awaji diagnostic criteria to diagnose ALS according to electrodiagnostic criteria into definite or probable. | Duration: NA  Clinical onset:  NA |
| Nodera [8] | Japan | Prospective cohort | 35 | 37 | 20/15 | 13/24 | 55.3 ± 8.3 | 58.0 ± 11.5 | 32.1 ± 12.9 | Cervical nerve root:  Longitudinally with coronal oblique plane. | LOGIQ7 (GE) with an 11-MHz linear array transducer. | Supine position. The maximal diameter and (CSA) were measured inside the hyperechoic rim (epineurium). | The revised El Escorial criteria and Awaji diagnostic criteria to diagnose ALS according to electrodiagnostic criteria into definite or probable.  ALS patients were assessed according to the ALS Functional Rating Scale-Revised (ALSFRS-R). | Duration: 2.9 ± 2.0 (years)  Clinical onset:  NA |
| Noto [25] | Australia | Prospective cohort | 53 | 30 | 41/12 | 20/10 | 59.9 ± 13.9 | 59.7 ± 17.6 | 39.5 ± 6.7 | The median and ulnar nerves were imaged at the wrist crease, the forearm, and the midpoint of the upper arm. | A MyLab Alpha (Esaote, Genova, Italy) was used with a 6-18 MHz broadband linear array transducer (SL2325, Esaote). | In a supine position for the median and ulnar nerves.  In prone position for the tibial nerves.  Both performed on the right side. | The Awaji electrodiagnostic criteria to classify the patients as definite (25) or probable (28) ALS.  Each patients underwent a neurological examination combined with direct investigations including electromyography EMG.  The ALS Functional Rating Scale-Revised (ALSFRS-R) to assess each ALS patient. | Duration: 23.0 ± 21.1  Clinical onset:  Bulbar: 22 (41)  Upper Limb:13 (25)  Lower limb: 18 (34) |
| Papadopoulou [26] | Greece | Case-control | 21 | 28 | 16/5 | 18/10 | 61 ± 11.07 | 57 ± 8.94 | 35.10 ± 9.8 | Vagus:  Bilaterally at the level of the thyroid gland. | Philips CX50 ultrasound system with a linear transducer at 12 Hz. | Participants were in the supine position and the sonographer behind them. | The ALS Functional Rating Scale (ALS-FRS) to evaluate the functional status of patients with ALS.  Composite autonomic symptom score (COMPASS 31) to assess and quantify autonomic symptoms severity across multiple autonomic domains. | Duration: 17.1 ± 16.98  Clinical onset:  LMN: 20 (95)  UMN: 20 (90)  Bulbar: 13 (62) |
| Diaz [27] | Spain | cross-sectional | 59 | 20 | 35/24 | 10/10 | 63.5 ± 10.89 | 60.2 ± 9.97 | 38.5 ± 5.44 | Median:  Midpoint of the arm between the medial epicondyle and the axilla | Canon Medical Systems Aplio XG (2008) with a 7– 13 MHz phased array transducer. | In supine position with the arm supinated and abducted beside the body. | The revised El Escorial criteria to recruit the patients as possible, probable, or definitive ALS.  The Amyotrophic Lateral Sclerosis Functional Rating Scale revised (ALSFRS-r) scale (0–48) to assess the disability. | Duration: 3.5 ± 6.25  Clinical onset:  Upper limb: 16 (27.1)  Lower limb: 28 (47.5)  Bulbar: 15 (25.4) |
| Schreiber 2015 [32] | Germany | Prospective cohort | 78 | 18 | 48/22 | 14/4 | 60.28 ± 12.62 | 63.8 ± 9.5 | 35.03 ± 6.36 | Visualized over the whole distance.  Ulnar:  From the distal wrist crease to the CuT.  Median:  From the distal wrist crease to the middle third of the forearm. | High-end ultrasound system (LOGIQ 7; GE Healthcare) with a 12-MHZ linear array probe. | Seated with the entire arm extended anteriorly, supinated, and supported at mid-thoracic height. | The diagnosis of ALS was based on the revised El Escorial criteria.  All patients were graded using the ALS Functional Rating Scale—Revised (ALSFRS-R). | Duration:  PLS: 104.1 ± 77.7  UMND: 32.8 ± 22.4  LMND: 37.0 ± 26.0  Classic: 20.9 ± 12.8  Bulbar: 15.7 ± 5.7  Clinical onset:  Classic: 21  UMND: 14  LMND: 20  Bulbar: 15  PLS: 8 |
| Schreiber 2018 [28] | Germany | Prospective cohort | 41 | 18 | 26/15 | 12/6 | 64 ± 10 | 59 ± 8 | 11 | Ulnar:  At the wrist and at the lower to middle third of the forearm.  Median:  At the mid-forearm and at the mid-humerus.  Radial:  At the forearm and at the posterior aspect of the humerus. | 12 MHz linear array probe (GE High-End LOGIQ®7 System). | In a seated position with the investigator facing the participant. | The El Escorial criteria to diagnose the patients as definite, probable, or possible ALS.  Overall disease severity was assessed using the revised ALS functional rating scale (ALSFRS-R). | Duration: 34 ± 38  Clinical onset:  Bulbar: 6 (15)  Upper limb: 20 (57)  Lower limb:15 (43) |
| Schreiber 2019 [29] | Germany | Retrospective cohort | 173 | 50 | 105/68 | 30/20 | 62 ± 11.5 | 60 ± 12 | 35.86 ± 6 | Ulnar:  At the wrist and at the lower to middle third of the forearm.  Median:  Mid-forearm at the mid-humerus. | 12 MHz linear array probe (GE High-End LOGIQ®7 System). | In seated position with the investigator facing them. | The revised El Escorial criteria to diagnose the included patients with definite, probable, laboratory-supported probable, or possible disease.  The revised ALS functional rating scale (ALSFRS-R) to evaluate overall disease severity. | Training cohort (61)  Duration: 25.93 ± 21.65  Clinical onset:  Bulbar: 10 (16)  Upper limb: 29 (48)  Lower limb: 22 (36)  Study cohort (112)  Duration:27.37 ± 26.91  Clinical onset:  Bulbar: 36 (32)  Upper limb: 42 (38)  Lower limb: 34 (30) |
| Schreiber 2020 [30] | Germany | Prospective cohort | 177 | 57 | 107/70 | 33/24 | 62.3 ± 11.6 | 59.4 ± 9.7 | 28.67 ± 7.89 | Median:  At the mid-forearm around 10 cm above the retinaculum flexorum.  Ulnar:  At the lower to middle third of the forearm. | 12 MHz linear array probe (GE Healthcare (Chicago, IL, USA) High-End LOGIQ®7 System). | In seated position with the investigator facing them. | The revised El Escorial criteria to diagnose the included patients with definite, probable, laboratory-supported probable, or possible disease.  Overall disease severity was assessed using the ALS Functional Rating Scale—Revised (ALSFRS-R) | Duration: 37.53 ± 22.99  Clinical onset:  Bulbar: 48(27)  Upper limb: 69(40)  Lower limb: 60(33) |
| Suratos [10] | Japan | Retrospective cohort | 38 | 28 | 23/15 | 10/18 | 64.71 ± 12.76 | 59.61 ± 24.45 | 38.41±6.87 | The phrenic nerve is measured as it crosses over the anterior scalene muscle, coursing towards the internal jugular vein and common carotid artery on the medial side of the neck. | 12-MHz linear array transducer. | Participants were assessed in supine position. | The updated Awaji criteria to diagnose the included patients with definite (9), probable (14), laboratory-supported probable(14), or possible disease (1).  The ALS Functional Rating Scale (ALS-FRS) to evaluate the functional status of patients with ALS. | Duration: 26.16 ± 23.11  Clinical onset:  Bulbar: 9(24)  Upper limb: 24(63)  Lower limb: 5(13) |
| Weise [31] | Germany | Case-control | 37 | 40 | 20/17 | 18/22 | 64.8 ± 11.4 | 65.7 ± 11.8 | 30.6 ± 6.0 | Vagus:  At the level of the thyroid gland.  Median:  10 cm proximal to the wrist crease. | The Esaote MyLab Five system with a 15 MHz transducer. | Offline using Viewpoint (5.6.25.281, General Electric Company) and in a blinded fashion. | The revised El Escorial criteria to recruit the patients with a clinical diagnosis of probable (n = 14) or definitive (n = 23).  The ALS Functional Rating Scale (ALS-FRS) to evaluate the functional status of patients with ALS | Duration: 22.5 ± 23.0  Clinical onset:  Bulbar: 18 (48.65%) |

Table S2. Quality assessment of the included studies according to Newcastle-Ottawa quality Scale (NOS) quality assessment tool for case control studies.

| study | Year | Selection | | | | Comparability | | Exposure | | | Score |
| --- | --- | --- | --- | --- | --- | --- | --- | --- | --- | --- | --- |
|  |  | adequate case definition | Representativeness of the cases | Selection of Controls | Definition of Controls | Study controls for age | Study controls for  any additional factor (sex, Edu, SE, dis..) | Ascertainment of exposure | Same method of ascertainment for cases and controls | Non-Response rate |  |
| Mohamed  et al. | 2021 | **★** | **★** | **-** | **★** | **★** | **★** | **-** | **-** | **-** | 5 |
| Papadopoulou et al. | 2022 | **★** | **★** | **-** | **-** | **★** | **★** | **-** | - | **-** | 4 |
| Cartwright et al. | 2011 | **★** | **-** | **★** | **★** | **★** | **★** | **-** | **★** | **-** | 6 |
| Weise et al. | 2022 | **★** | **-** | **-** | **★** | **★** | **★** | **-** | **★** | - | 5 |
| Holzapfel et al. | 2020 | **★** | **★** | **-** | - | **★** | - | - | - | - | 3 |
| Mori et al. | 2014 | **★** | - | **-** | **★** | - | - | - | - | - | 2 |

| Study | Year | C. 1 | C. 2 | C. 3 | C. 4 | C. 5 | C. 6 | C. 7 | C. 8 | C. 9 | C. 10 | C. 11 | C. 12 | C. 13 | C. 14 | Total score |
| --- | --- | --- | --- | --- | --- | --- | --- | --- | --- | --- | --- | --- | --- | --- | --- | --- |
| Martínez payáa | **2022** | YES | NO | YES | YES | NO | NO | NO | NO | YES | YES | YES | YES | NO | YES | **8** |
| Suratos | **2021** | YES | YES | YES | YES | NO | NO | NO | NO | YES | NO | YES | YES | NO | NO | **7** |
| Schreiber | **2020** | YES | YES | YES | YES | YES | NO | NO | NO | YES | NO | YES | NO | NO | NO | **7** |
| Schreiber | **2019** | YES | YES | YES | YES | NO | NO | NO | YES | YES | NO | YES | NO | NO | NO | **7** |
| Deilami | **2019** | YES | YES | YES | YES | NO | NO | NO | NO | YES | NO | YES | YES | NO | NO | **7** |
| Díaz | **2019** | YES | NO | YES | YES | YES | NO | NO | NO | YES | NO | YES | YES | NO | YES | **8** |
| Schreiber | **2018** | YES | YES | YES | YES | NO | NO | NO | YES | YES | NO | YES | NO | NO | NO | **7** |
| Noto | **2018** | YES | YES | YES | YES | NO | NO | NO | YES | YES | NO | YES | YES | NO | NO | **8** |
| Grimm | **2015** | YES | YES | YES | YES | NO | NO | NO | YES | YES | YES | YES | YES | NO | NO | **9** |
| Schreiber | **2015** | YES | YES | YES | YES | NO | NO | NO | YES | YES | YES | YES | NO | NO | NO | **8** |
| Nodera | **2014** | YES | NO | YES | YES | NO | NO | NO | NO | YES | NO | YES | YES | NO | NO | **6** |

Table S3. **Quality assessment of the included studies according to the National Institute of Health**

**(NIH) quality assessment tool for observational cohort and cross-sectional studies.**

| Was the research question or objective in this paper clearly stated? | Criterion 1 |
| --- | --- |
| Was the study population clearly specified and defined? | Criterion 2 |
| Was the participation rate of eligible persons at least 50%? | Criterion 3 |
| Criterion 4 Were all the subjects selected or recruited from the same or similar populations (including the same time period)? Were inclusion and exclusion criteria for being in the study prespecified and applied uniformly to all participants? | Criterion 4 |
| Was a sample size justification, power description, or variance and effect estimates provided? | Criterion 5 |
| For the analyses in this paper, were the exposure(s) of interest measured prior to the outcome(s) being measured? | Criterion 6 |
| Was the timeframe sufficient so that one could reasonably expect to see an association between exposure and outcome if it existed? | Criterion 7 |
| For exposures that can vary in amount or level, did the study examine different levels of the exposure as related to the outcome (e.g., categories of exposure, or exposure measured as continuous variable)? | Criterion 8 |
| Were the exposure measures (independent variables) clearly defined, valid, reliable, and implemented consistently across all study participants? | Criterion 9 |
| Was the exposure(s) assessed more than once over time? | Criterion 10 |
| Were the outcome measures (dependent variables) clearly defined, valid, reliable, and implemented consistently across all study participants? | Criterion 11 |
| Were the outcome assessors blinded to the exposure status of participants? | Criterion 12 |
| Was loss to follow-up after baseline 20% or less? | Criterion 13 |
| Were key potential confounding variables measured and adjusted statistically for their impact on the relationship between exposure(s) and outcome(s)? | Criterion 14 |

**Table S4. Mean difference of CSA values for nerves reported by single studies.**

|  | | **Controls** | | | **ALS** | | |  |  |
| --- | --- | --- | --- | --- | --- | --- | --- | --- | --- |
| **Study** | **Nerve measured** | **Mean**  (mm^2^) | **SD** | **Number** | **Mean**  (mm^2^) | **SD** | **Number** | **MD**  (mm^2^) | **P** |
| Nodera [8] | C6 root | 7.6 | 1.6 | 37 | 5.34 | 1.6 | 35 | 2.26 | <0.001 |
| Suratos [10] | Phrenic (Rt) | 1.62 | 0.45 | 28 | 1.08 | 0.39 | 38 | 0.54 | <0.00001 |
|  | Phrenic (Lt) | 1.39 | 0.31 | 28 | 1.02 | 0.34 | 38 | 0.37 | <0.00001 |


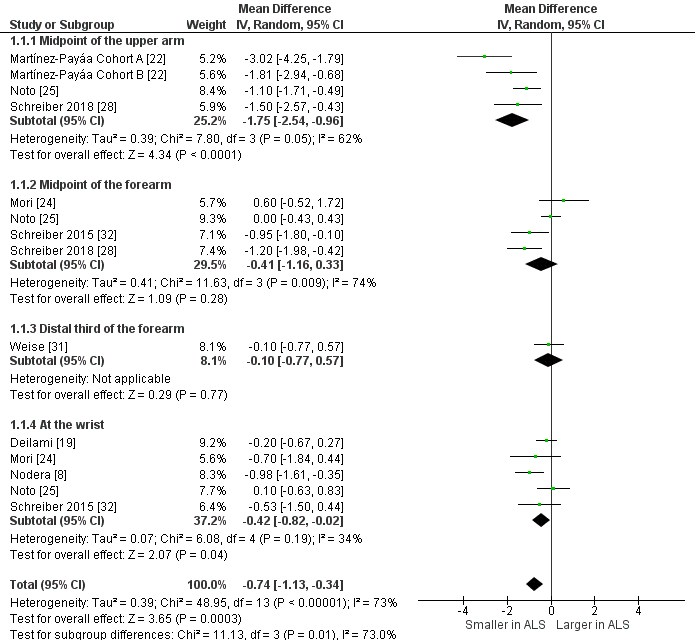


Figure S1. Mean difference of right median nerve cross sectional area between ALS patients and healthy controls.

ALS: Amyotrophic Lateral Sclerosis, SD: Standard deviation, CI: Confidence Interval

*Cohort A comprised ALS patients diagnosed more than 6 months

*Cohort B comprised ALS patients with a recent (within 3 months) ALS diagnosis


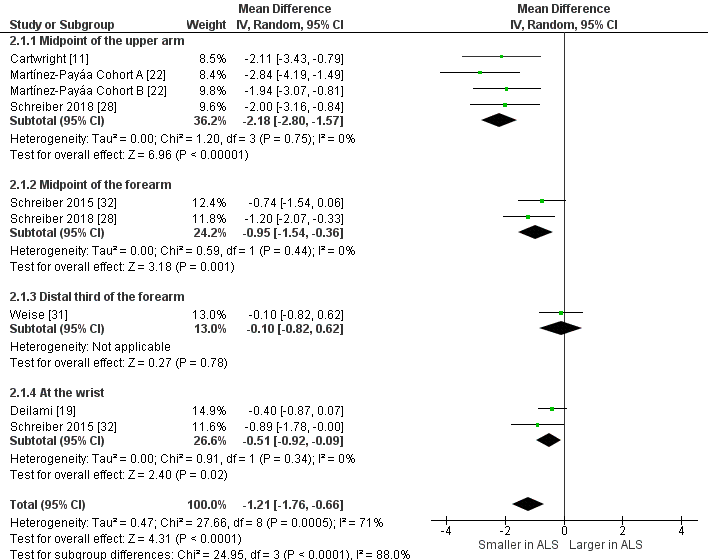


Figure S2. Mean difference of left median nerve cross sectional area between ALS patients and healthy controls.

ALS: Amyotrophic Lateral Sclerosis, SD: Standard deviation, CI: Confidence Interval

*Cohort A comprised ALS patients diagnosed more than 6 months

*Cohort B comprised ALS patients with a recent (within 3 months) ALS diagnosis


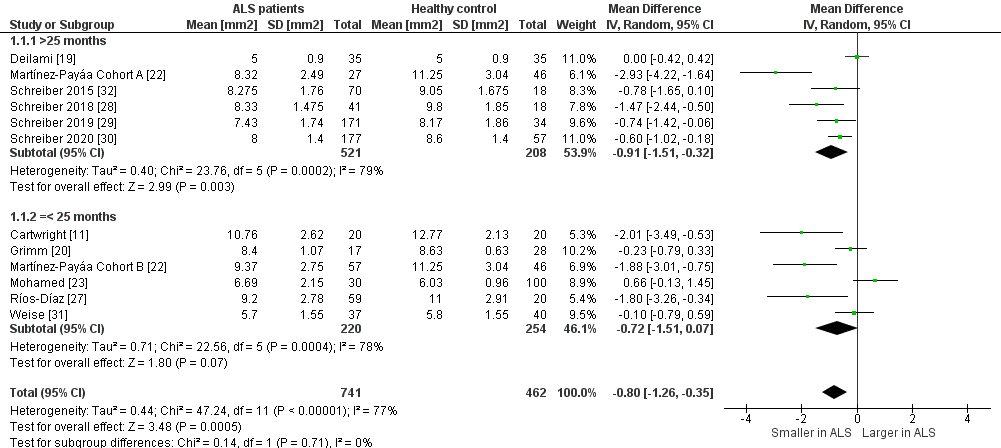


Figure S3. Subgroup analysis by ALS disease duration for median nerve group


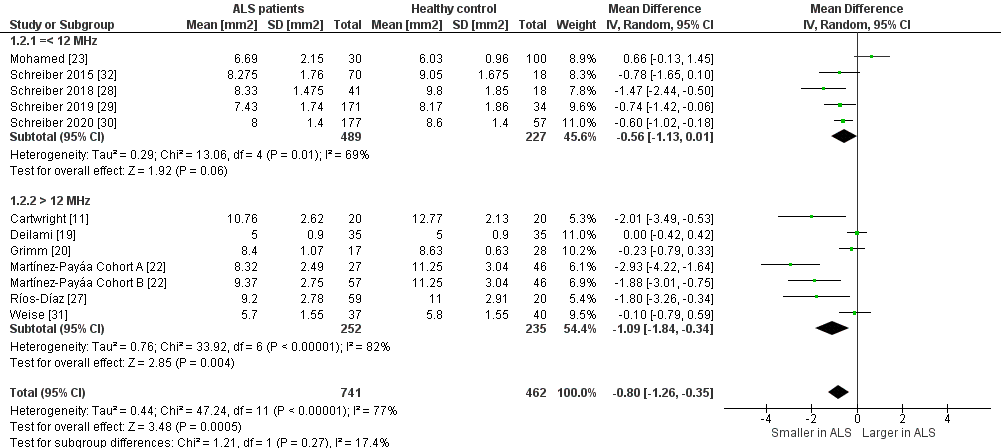


Figure S4. Subgroup analysis by ultrasound probe frequency for median nerve group


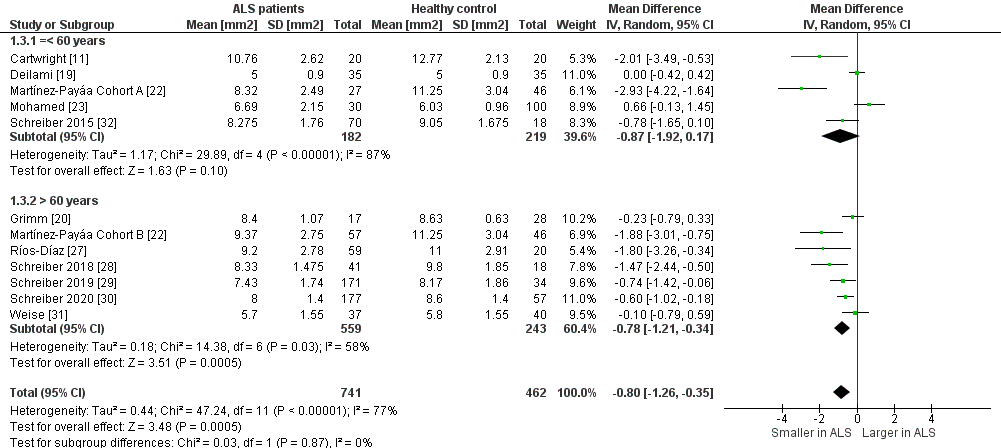


Figure S5. Subgroup analysis by age for median nerve patients


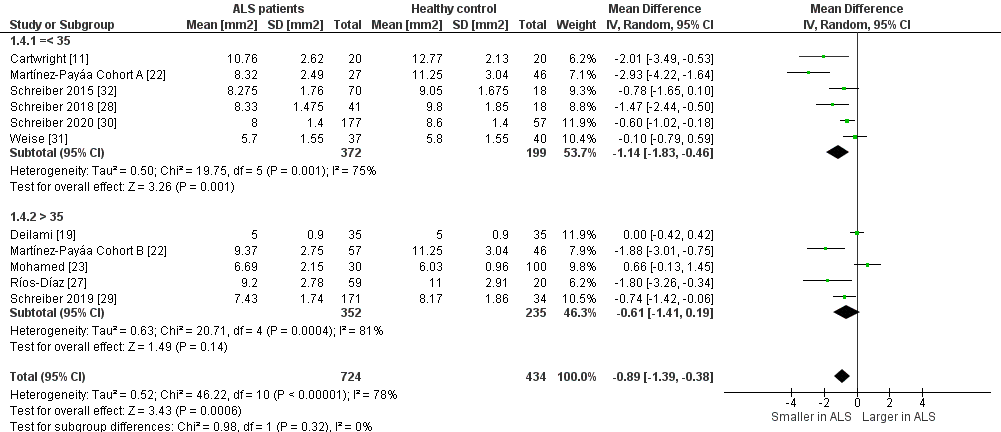


Figure S6. Subgroup analysis by ALSFR for median nerve patients


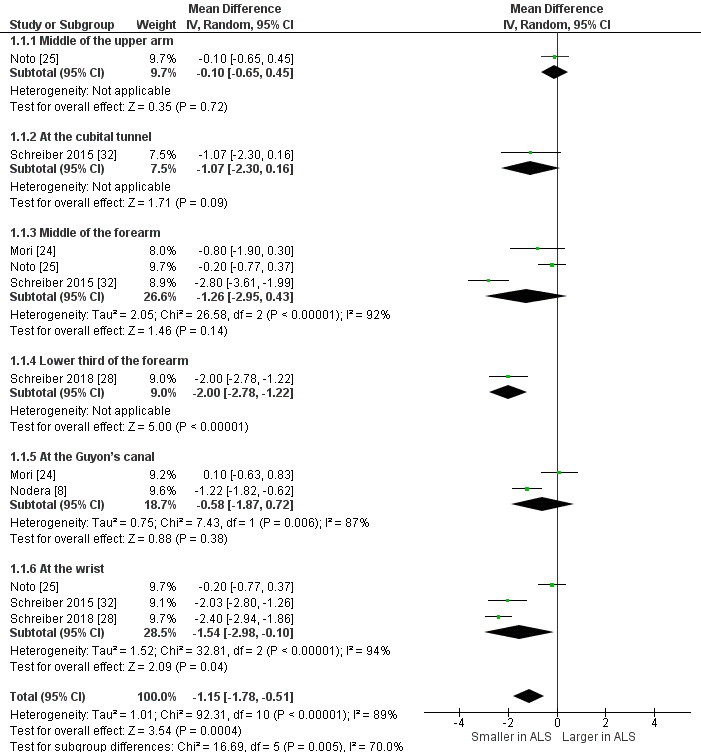


Figure S7. Mean difference of right ulnar nerve cross sectional area between ALS patients and healthy controls.

ALS: Amyotrophic Lateral Sclerosis, SD: Standard deviation, CI: Confidence Interval


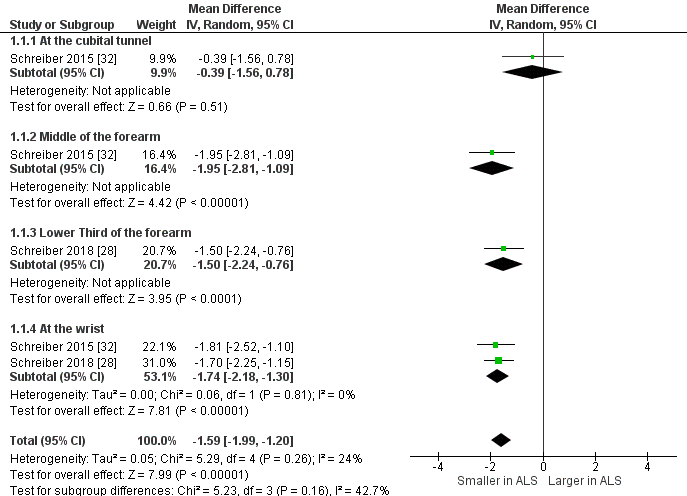


Figure S8. Mean difference of left ulnar nerve cross sectional area between ALS patients and healthy controls.


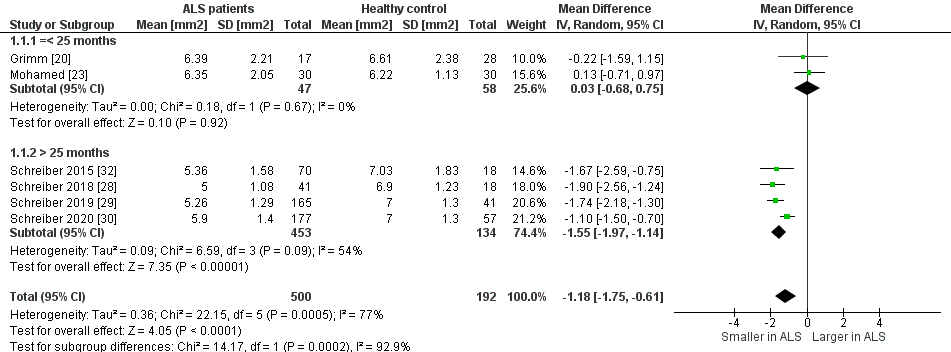


Figure S9. Subgroup analysis by disease duration for ulnar nerve group


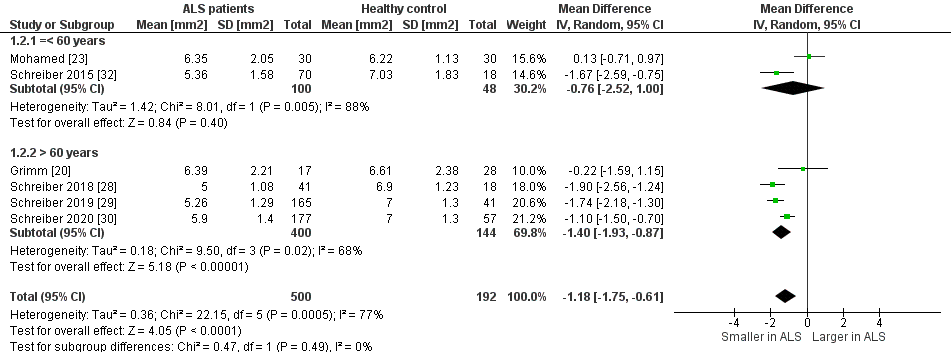


Figure S10. Subgroup analysis by Age for ulnar nerve patients


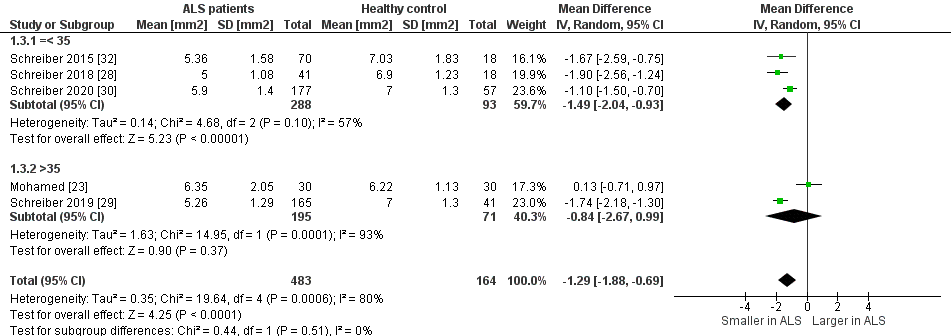
Figure S11. Subgroup analysis by ALSFR for ulnar nerve patients


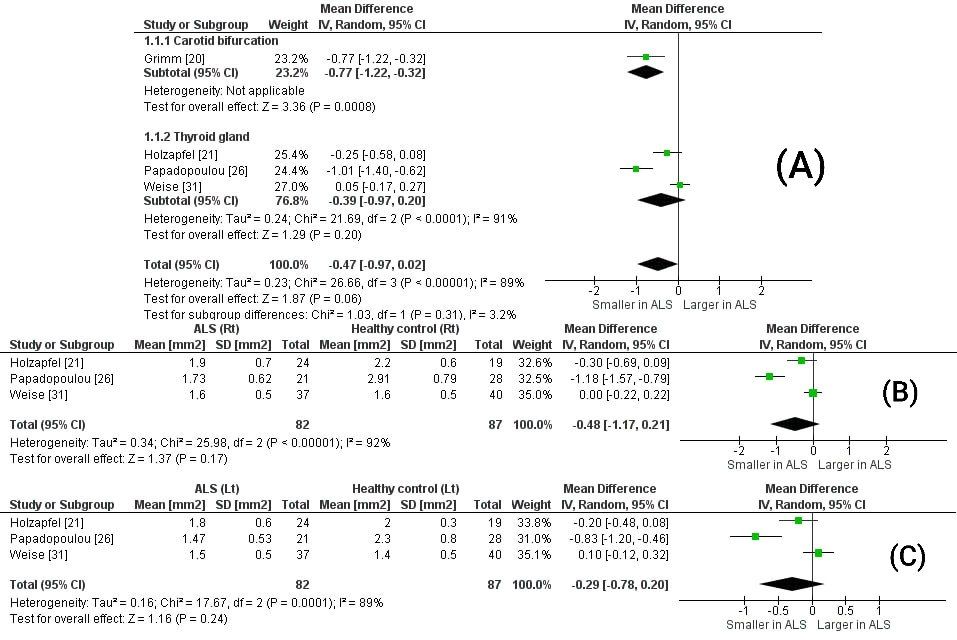


Figure S12. Mean difference of vagus nerve cross sectional area between ALS patients and healthy controls.

1. Average bilateral vagal nerve
2. Right vagal nerve
3. Left vagal nerve


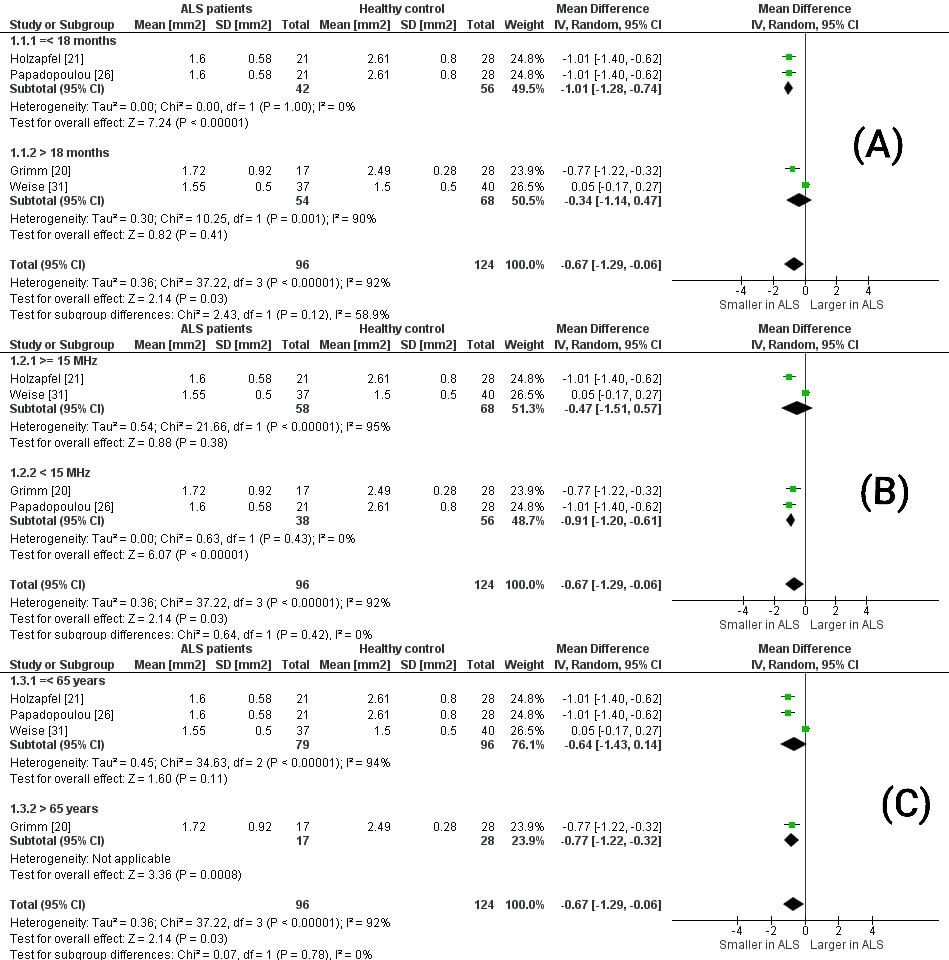
Figure S13. Subgroup analysis for vagus nerve patients

1. By disease duration
2. By US probe
3. By age


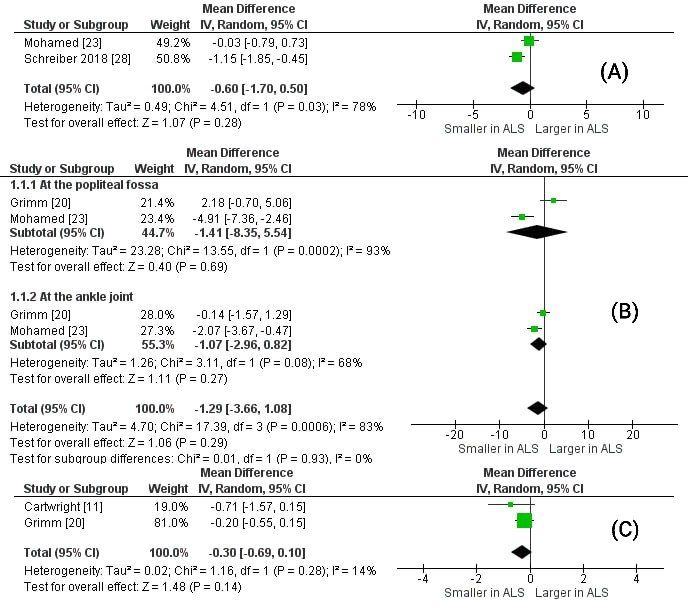


Figure S14.

1. Mean difference of **radial** nerve cross-sectional area between ALS patients and healthy controls
2. Mean difference of **tibial** nerve cross-sectional area between ALS patients and healthy controls
3. Mean difference of **sural** nerve cross-sectional area between ALS patients and healthy controls


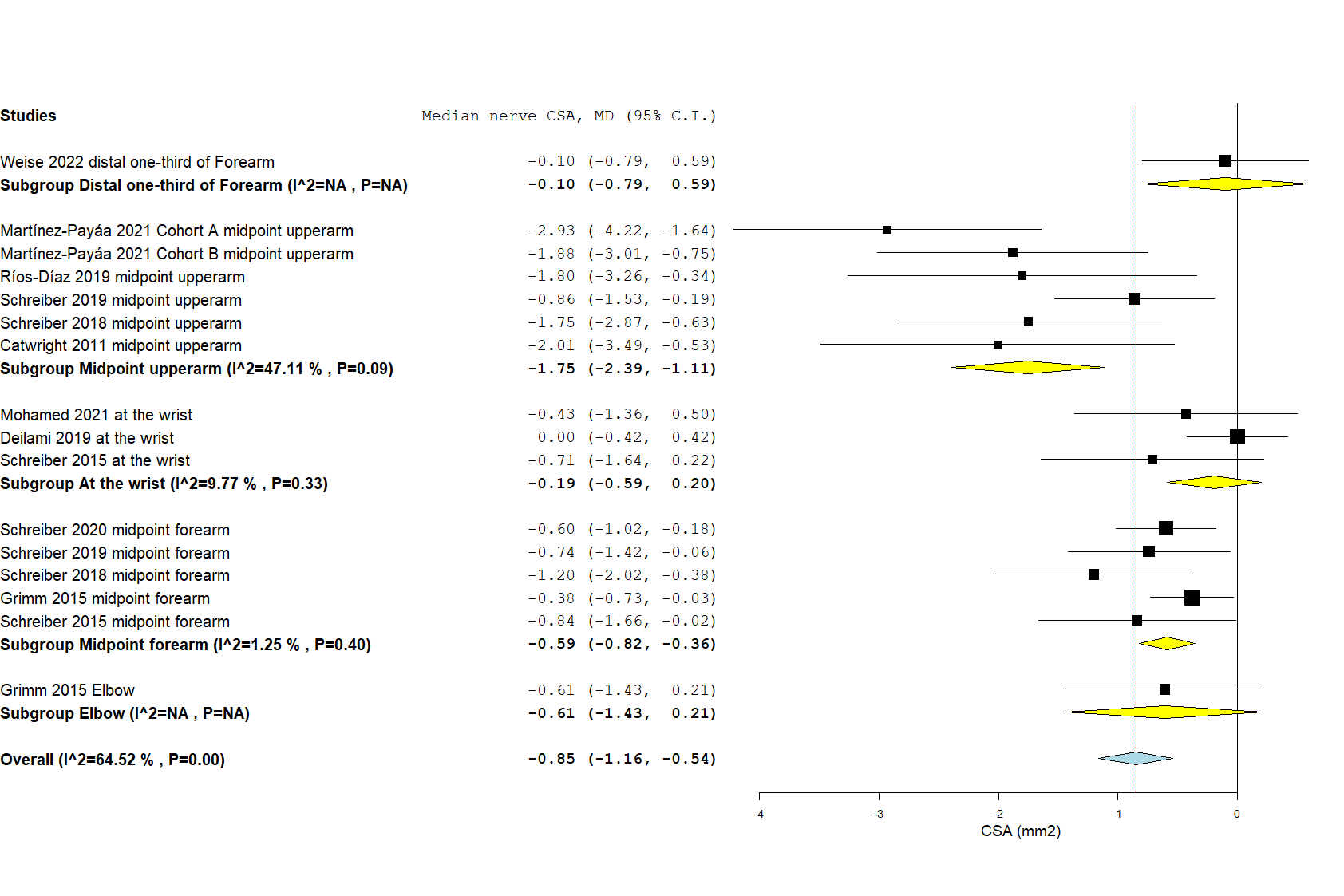
Figure S15. Sensitivity analysis of median nerve (Mohamed2021 and Grimm2015 out)


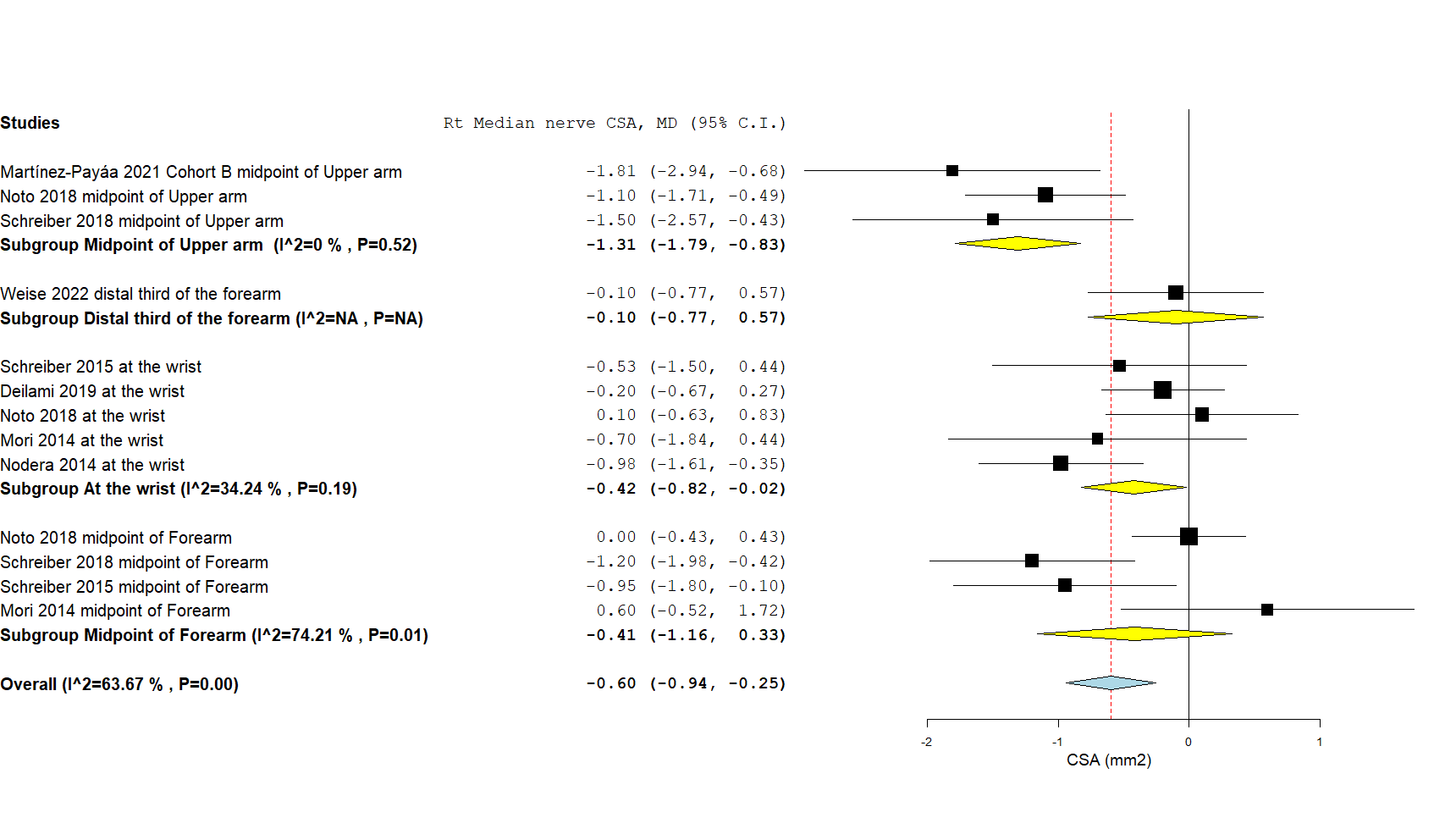
Figure S16. Sensitivity analysis of right median nerve (Martínez-Payáa 2022 cohort A out)


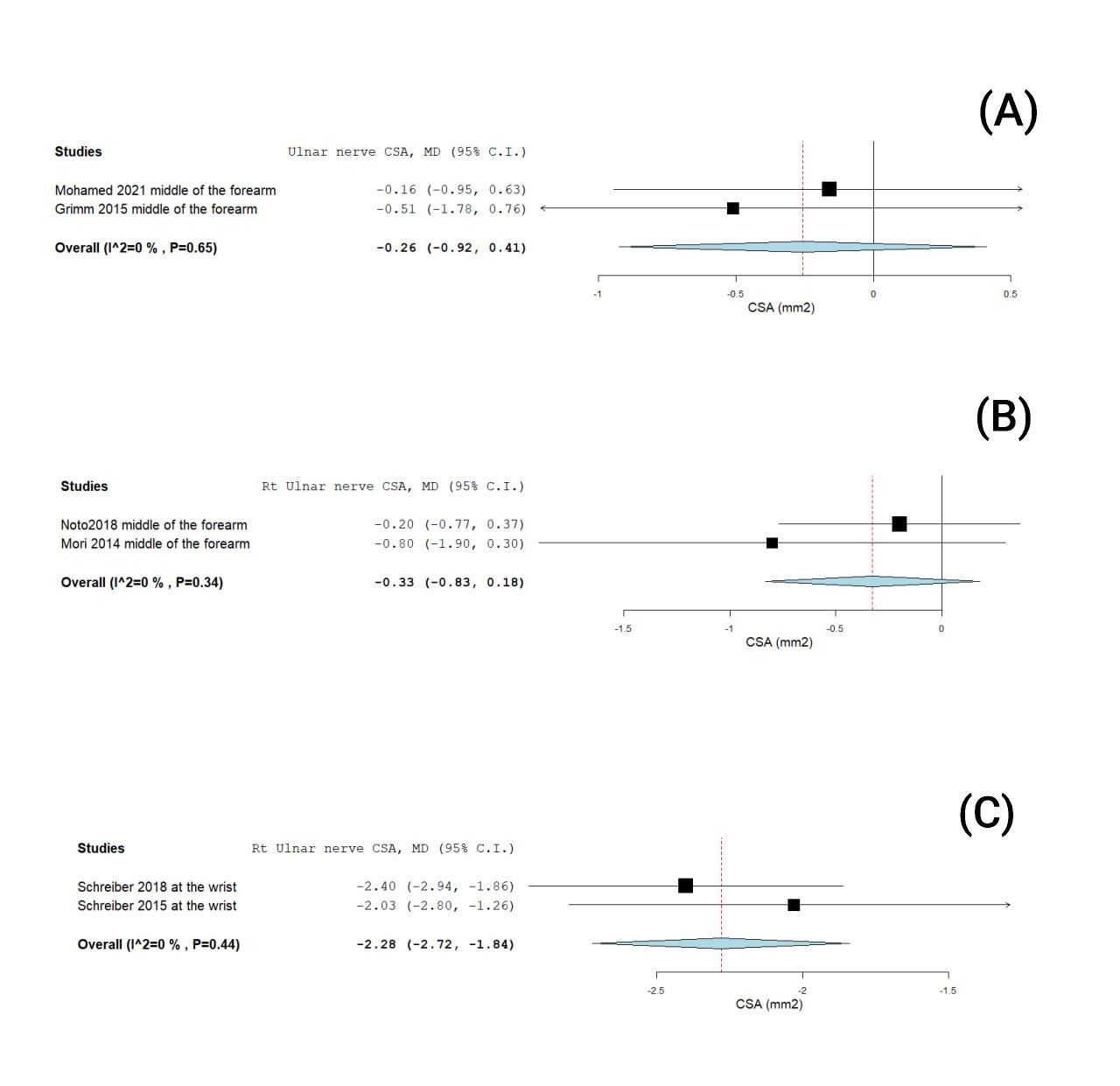


Figure S17

(A). Sensitivity analysis of bilateral ulnar nerve (Schreiber et al. 2015 out)

(B). Sensitivity analysis of right ulnar nerve (Schreiber et al. 2015 out)

(C). Sensitivity analysis of right ulnar nerve (Noto 2018 out)
